# Fate of arsenicals in mice carrying the human *AS3MT* gene exposed to environmentally relevant levels of arsenite in drinking water

**DOI:** 10.1038/s41598-023-30723-8

**Published:** 2023-03-04

**Authors:** Christelle Douillet, Madison Miller, Peter H. Cable, Qing Shi, Hisham El-Masri, Tomáš Matoušek, Beverly H. Koller, David J. Thomas, Miroslav Stýblo

**Affiliations:** 1 Department of Nutrition, Gillings School of Global Public Health, University of North Carolina at Chapel Hill, Chapel Hill, NC 27599-7461 USA; 2 Chemical Characterization and Exposure Division, Center for Computational Toxicology & Exposure, Office of Research and Development, U.S. Environmental Protection Agency, Research Triangle Park, NC 27709 USA; 3 Institute of Analytical Chemistry of the Czech Academy of Sciences, v. v. i., Veveří 97, 602 00 Brno, Czech Republic; 4 Department of Genetics, School of Medicine, University of North Carolina, Chapel Hill, NC 27599 USA; 5 Dinkey Creek Consulting, LLC, Chapel Hill, NC 27517 USA

**Keywords:** Genetics, Environmental sciences, Natural hazards, Risk factors

## Abstract

Although mice are widely used to study adverse effects of inorganic arsenic (iAs), higher rates of iAs methylation in mice than in humans may limit their utility as a model organism. A recently created 129S6 mouse strain in which the *Borcs7/As3mt* locus replaces the human *BORCS7/AS3MT* locus exhibits a human-like pattern of iAs metabolism. Here, we evaluate dosage dependency of iAs metabolism in humanized (Hs) mice. We determined tissue and urinary concentrations and proportions of iAs, methylarsenic (MAs), and dimethylarsenic (DMAs) in male and female Hs and wild-type (WT) mice that received 25- or 400-ppb iAs in drinking water. At both exposure levels, Hs mice excrete less total arsenic (tAs) in urine and retain more tAs in tissues than WT mice. Tissue tAs levels are higher in Hs females than in Hs males, particularly after exposure to 400-ppb iAs. Tissue and urinary fractions of tAs present as iAs and MAs are significantly greater in Hs mice than in WT mice. Notably, tissue tAs dosimetry in Hs mice resembles human tissue dosimetry predicted by a physiologically based pharmacokinetic model. These data provide additional support for use of Hs mice in laboratory studies examining effects of iAs exposure in target tissues or cells.

## Introduction

Chronic exposure to inorganic arsenic (iAs) through ingestion of contaminated water and food^1,2^ is a serious public health concern in the U.S. and worldwide. Chronic exposures to iAs have been linked to cancers^3^ and other potentially fatal diseases^1,4^. A central feature of iAs metabolism is methylation of iAs that is catalyzed by arsenic (+ 3 oxidation state) methyltransferase (AS3MT). This enzyme uses methyl groups from S-adenosylmethionine to convert iAs to monomethyl-arsenic (MAs) and dimethyl-arsenic (DMAs) metabolites^5^. Paradoxically, methylation of iAs creates unique methylated arsenicals which mediate some of the toxic effects associated with iAs exposure and also detoxifies iAs through the formation of rapidly excreted methylated species^6,7^. The efficiency of iAs methylation, which is typically characterized by proportions of urinary arsenic excreted as MAs and DMAs, also plays a key role in susceptibility to adverse effects of iAs exposure. Individuals with higher percentages of total arsenic (tAs) in urine present as iAs (%iAs) or MAs (%MAs), and a lower percentage of DMAs (%DMAs), or with a lower DMAs/MAs ratio in urine are at a higher risk of developing cancer or other diseases associated with chronic iAs exposure^8–11^.

In laboratory studies, mice have been used to examine iAs metabolism and to study associations between iAs exposure and the pathogenesis of many diseases. These studies have provided information about cellular and molecular targets of iAs exposure and elucidated mechanisms underlying the toxic and carcinogenic effects of iAs. However, there are significant differences in patterns and extent of iAs methylation in humans and mice. Overall, mice methylate iAs more efficiently than do humans and this higher rate of methylation is associated with faster rates of whole body and urinary clearance of arsenic in mice than in humans^12,13^. Between-species differences in patterns for methylation and excretion of arsenic are well-illustrated by differences in patterns of urinary metabolites after iAs exposure. In iAs-treated mice, only trace amounts of MAs and iAs are found in urine and DMAs is the major urinary metabolite, accounting for over 90% of tAs^14^. In contrast, for humans using water supplies containing up to about 1000-parts per billion (ppb) iAs, urine typically contains 10–30% iAs, 10–20% MAs, and 60–70% DMAs^12,15,16^. These differences between species in iAs methylation rate are reflected by differences in tissue arsenic concentrations. Thus, for mice and humans exposed to the same level of iAs in drinking water, a physiologically based pharmacokinetic (PBPK) model for iAs retention predicted tAs concentrations in human tissues to be up to an order of magnitude higher than those in tissues of mice^17,18^. Similarly, tissue levels and proportions of methylated metabolites, which have different toxicities and modes of actions, may also differ between humans and mice. These interspecies differences in iAs metabolism represent a serious challenge for laboratory studies and may confound efforts to translate results obtained in mouse-based studies to humans.

Our earlier work focused on developing a mouse model in which iAs metabolism resembled that found in humans. Studies of iAs metabolism in 12 genetically diverse mouse strains produced by the Collaborative Cross (CC), a large panel of recombinant inbred mouse lines developed to analyze human phenotypes with complex etiologies produced by interactions between allele combinations and the environment^19^, found only minor differences in iAs methylation capacity in CC strains exposed to 0.1 and 50-parts per million (ppm) iAs in drinking water^14^. At both iAs exposure levels, DMAs accounted for 96–99% of tAs in urine from all CC strains, suggesting that capacity to methylate iAs in these genetically diverse mice greatly exceeded that in humans. Notably, in livers from CC mice, *As3mt* expression correlated positively with %DMAs and negatively with %iAs, thus supporting previously published findings that AS3MT is the key enzyme in the pathway for methylation of iAs^20,21^.

The arsenic methyltransferases in the human and mouse genomes share only 75% of the primary sequence^22^. To explore the hypothesis that the inter-species differences in capacity to convert iAs into its methylated metabolites are related to differences in the structure and function of these arsenic methyltransferases, we generated a variant of the 129S6 mouse strain in which the *As3mt* gene and the adjacent *Borcs7* gene were humanized by syntenic replacement^18^. In this earlier work, we examined iAs metabolism in this humanized (Hs) mouse strain after a single dose of arsenite and during a sub-chronic exposure to 400-ppb iAs (as arsenite) in drinking water. In these Hs mice, the pattern of iAs metabolism, including the proportions of arsenic metabolites in urine, closely resembled iAs metabolism in humans. However, this earlier work provided only limited data on tissue disposition of arsenic metabolites and only after the exposure to 400-ppb iAs^18^. Here, we extended this work with additional information on concentrations and proportions of arsenic metabolites in tissues of Hs mice after exposure to 400-ppb iAs (Study 1) and also the disposition of arsenic metabolites in Hs mice after exposure to a lower iAs concentration, 25-ppb (Study 2).

The present research compared patterns of tissue distribution and urinary excretion of iAs and its methylated metabolites in Hs and wild-type (WT) 129S6 mice. We found that iAs metabolism in Hs mice exposed to two sub part-per-million concentrations of iAs in drinking water was characterized by low efficiency of iAs methylation, resulting in a lower urinary excretion of arsenic and higher retention of arsenic species in tissues. At both exposure levels, the proportions of urinary arsenic species in Hs mice resembled those reported for human urine, suggesting that this mouse strain may serve as an appropriate model to study adverse effects of iAs exposure in laboratory settings.

## Results

This research used Hs and WT mice to evaluate the contribution of arsenic methyltransferase genotype to interspecies differences in iAs metabolism. Within-genotype differences in arsenic methylation efficiency compared patterns of arsenic metabolism, distribution, and retention in male and female mice of the *As3mt* (WT) or *AS3MT* (Hs) genotypes. Between-genotype differences in arsenic methylation efficiency examined the effects of expression of *As3mt* or *AS3MT* in mice on the patterns of arsenic metabolism, distribution, and retention.

### Genotypic differences in concentrations and proportions of arsenic species in urine

**Figure 1:**
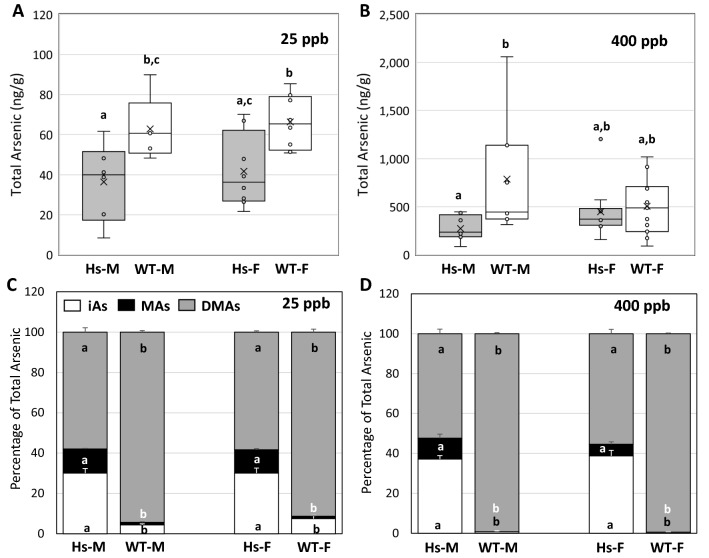
Total arsenic concentrations (**A**, **B**) and proportions of arsenic species (**C**, **D**) in urine of humanized (Hs) and wild-type (WT) male (M) and female (F) mice after 4-week exposure to 25-ppb (**A**, **C**) or 400-ppb (**B**, **D**) of inorganic arsenic in drinking water. For total arsenic concentrations (**A**, **B**), mean (×), median (—), 25th and 75th percentiles (box), maximum and minimum (whiskers), and individual values including outliers are shown. For the proportions of arsenic species (**C**, **D**), each bar and whisker represent mean and standard error of the mean (SEM) value, respectively. For 25-ppb exposure, N = 6 for Hs males and WT males, and N = 8 for Hs females and WT females. For 400-ppb exposure, N = 7 and 8 for Hs males and WT males, respectively, and N = 10 and 11 for Hs females and WT females, respectively. In panels (**A**, **B**), values marked with different letters are significantly different (p < 0.05). In panels (**C**, **D**), for each arsenic species, values marked with different letters are significantly different (p < 0.05).

Because urine is the main route for the excretion of iAs and its methylated metabolites in mice and humans exposed to iAs^23–28^, we examined between-genotype differences in urinary tAs levels in Hs and WT mice. For male and female mice exposed to 25-ppb iAs in drinking water, the Hs genotype was associated with significantly lower concentrations of tAs in urine than were found in WT mice (Fig. 1A). For both genotypes, there was no statistically significant difference in urinary tAs concentration of male and female mice. After exposure to 400-ppb iAs, urinary tAs levels were lower in Hs mice than in WT mice, although the difference was statistically significant only for Hs males and WT males; (Fig. 1B).

At both exposure levels, percentages of arsenic species in urine differed significantly between genotypes (Fig. 1C,D). Both iAs and MAs accounted for significantly larger fractions of urinary tAs in Hs mice than in WT mice. Notably, DMAs represented > 91% of tAs in urine of WT mice, but < 59% in urine of Hs mice. For each exposure group, proportions of arsenic species in urine of male and female mice of the same genotype did not differ significantly. There were significant differences in proportions of urinary arsenic species between mice exposed to 25 and 400-ppb iAs. For the Hs genotype, urinary %iAs was significantly higher in males and females exposed to 400-ppb iAs than in mice exposed to 25-ppb iAs (p = 0.028 and 0.038, respectively). Across exposure levels, statistically significant differences in %MAs were found; urinary %MAs was higher in Hs males and females exposed to 25-ppb than in the 400-ppb group (p = 0.001 and < 0.0001, respectively). Similarly, urinary %DMAs was lower in the 400-ppb group, although this difference was not statistically significant. For the WT genotype, urinary %DMA was significantly higher (p < 0.0001) in the 400-ppb group (> 99.2%) than in the 25-ppb group (91.3% for females and 94.3% for males). Both urinary %iAs and %MAs were significantly lower (p < 0.012) in WT male and female mice exposed to 400-ppb than in the 25-ppb group.

### Concentrations of tAs in tissues of WT and Hs mice

**Figure 2:**
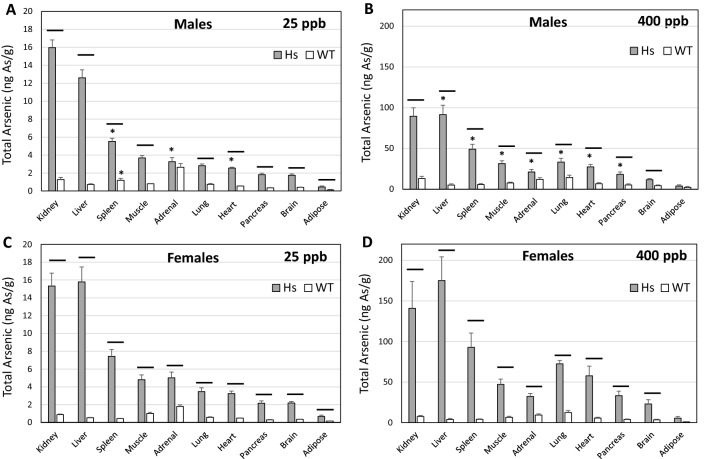
Concentrations of total arsenic in tissues of humanized (Hs) and wild-type (WT) male and female mice after 4-week exposure to 25-ppb (**A**, **C**) or 400-ppb (**B**, **D**) of inorganic arsenic in drinking water. Each bar and whisker represent mean and SEM value, respectively. For 25-ppb exposure, N = 6 for Hs males and WT males, and N = 8 for Hs females and WT females. For 400-ppb exposure, N = 7 and 8 for Hs males and WT males, respectively, and N = 10 and 11 for Hs females and WT females, respectively. Horizontal line marks significant difference between genotypes for individual tissue; asterisk marks significant difference between males and females of the same genotype (p < 0.05).

For both iAs exposure levels, tissue tAs concentrations, the sum of tissue concentrations of iAs, MAs, and DMA, were significantly higher in Hs mice than in WT mice (Fig. 2). Only in adrenal glands of male mice exposed to 25-ppb iAs did the difference between genotypes fail to reach statistical significance (Fig. 2A). At both exposure levels, in male and female Hs mice highest tAs levels were found in kidneys, liver, and spleen; lowest tAs concentration was found in adipose tissue. At both exposure levels, tissue tAs levels were higher in female Hs mice than in male Hs mice. In Hs mice exposed to 25-ppb iAs, these differences reached statistical significance in spleen, adrenal glands, and heart (Fig. 2A,C). In the 400-ppb iAs exposure group, the differences in tAs levels in liver, spleen, skeletal muscle, adrenal glands, lung, heart, and pancreas were statistically significant (Fig. 2B,D).

In WT mice exposed to 25-ppb iAs, tAs concentration was highest in adrenal glands. In the 400-ppb iAs exposure group, tAs concentrations were highest in lung, kidney, and adrenal glands. The only statistically significant difference in tAs concentrations in male and female WT mice occurred in spleens in the 25-ppb iAs exposure group (Fig. 2A). In the 400-ppb iAs exposure group, tissue tAs concentration in WT males and females did not differ significantly.

### Concentrations of iAs, MAs and DMAs in tissues of WT and Hs mice

**Figure 3:**
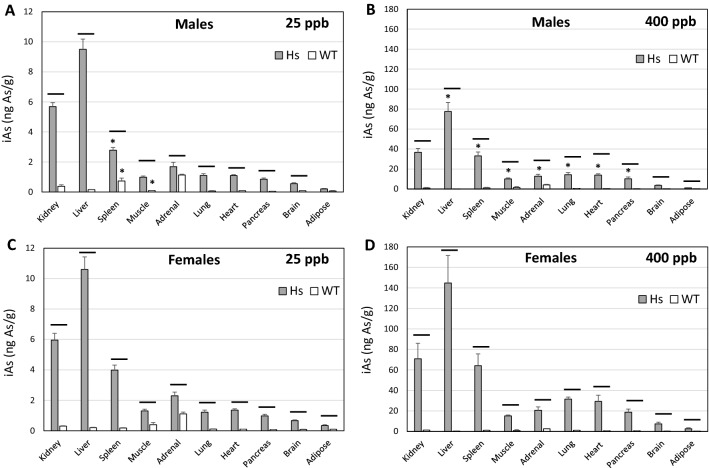
Concentrations of iAs in tissues of humanized (Hs) and wild-type (WT) male and female mice after 4-week exposure to 25-ppb (**A**, **C**) or 400-ppb (**B**, **D**) of inorganic arsenic in drinking water. Each bar and whisker represent mean and SEM value, respectively. For 25-ppb exposure, N = 6 for Hs males and WT males, and N = 8 for Hs females and WT females. For 400-ppb exposure, N = 7 and 8 for Hs males and WT males, respectively, and N = 10 and 11 for Hs females and WT females, respectively. Horizontal line marks significant difference between genotypes for individual tissue; asterisk marks significant difference between males and females of the same genotype (p < 0.05).

**Figure 4:**
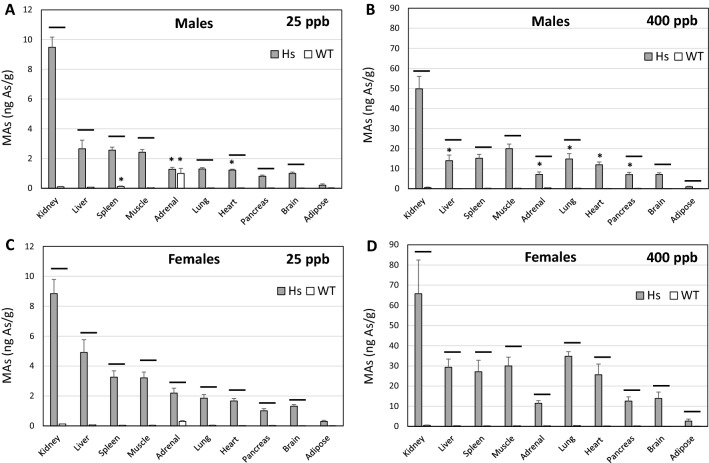
Concentrations of MAs in tissues of humanized (Hs) and wild-type (WT) male and female mice after 4-week exposure to 25-ppb (**A**, **C**) or 400-ppb (**B**, **D**) of inorganic arsenic in drinking water. Each bar and whisker represent mean and SEM value, respectively. For 25-ppb exposure, N = 6 for Hs males and WT males, and N = 8 for Hs females and WT females. For 400-ppb exposure, N = 7 and 8 for Hs males and WT males, respectively, and N = 10 and 11 for Hs females and WT females, respectively. Horizontal line marks significant difference between genotypes for individual tissue; asterisk marks significant difference between males and females of the same genotype (p < 0.05).

**Figure 5:**
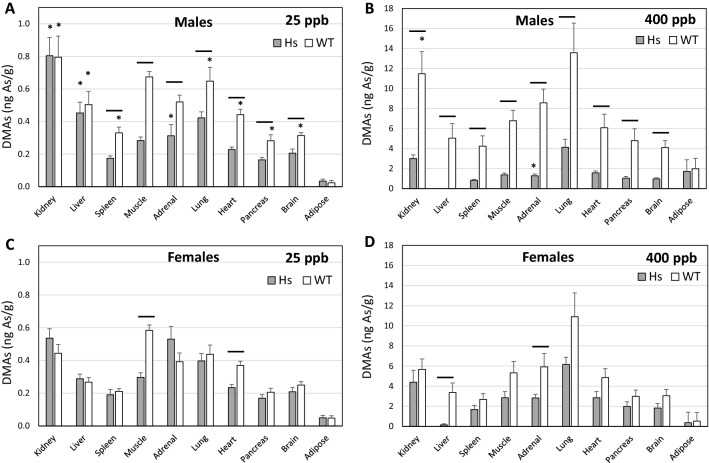
Concentrations of DMAs in tissues of humanized (Hs) and wild-type (WT) male and female mice after 4-week exposure to 25-ppb (**A**, **C**) or 400-ppb (**B**, **D**) of inorganic arsenic in drinking water. Each bar and whisker represent mean and SEM value, respectively. For 25-ppb exposure, N = 6 for Hs males and WT males, and N = 8 for Hs females and WT females. For 400-ppb exposure, N = 7 and 8 for Hs males and WT males, respectively, and N = 10 and 11 for Hs females and WT females, respectively. Horizontal line marks significant difference between genotypes for individual tissue; asterisk marks significant difference between males and females of the same genotype (p < 0.05).

Measurement of iAs, MAs, and DMAs in tissues provided additional insights into the relation between genotype and capacity for iAs methylation (Figs. 3, 4, 5). Statistically significant correlations were found between tAs concentrations and concentrations of iAs and MAs in tissues of both Hs and WT mice (Figs. S1 and S2). Notably, there were both between- and within-genotype differences in tissue levels of iAs and MAs of Hs and WT mice. Between-genotype comparisons found that iAs and MAs levels were higher in Hs mice than in WT mice and patterns of tissue concentrations of iAs and MAs differed between genotypes. In Hs mice in either iAs exposure group, iAs levels were highest in liver and MAs levels were highest in kidneys. In WT mice in either exposure group, highest iAs levels were found in adrenal glands. At the 25-ppb exposure level, MAs concentrations were highest in adrenal glands of WT mice. At the 400-ppb exposure level, MAs levels were highest in kidneys. Within-genotype comparisons for Hs mice found that iAs levels in spleen of females was significantly higher than in males in the 25-ppb exposure group. In the 400-ppb exposure group, iAs levels in all tissues except kidney, brain, and adipose were significantly higher in females than in males. Within-genotype comparisons for WT mice in the 25-ppb iAs exposure group found spleen iAs levels were significantly higher in males than females; conversely, iAs levels in muscle were significantly higher in females than in males. Within-genotype comparisons of tissue MAs levels in Hs mice found significantly higher levels in adrenal glands and heart of females than in males in the 25-ppb iAs exposure group. In the 400-ppb iAs exposure group, MAs levels in liver, adrenal glands, lung, and pancreas were significantly higher in females than in males. Similar within-genotype comparisons for WT mice found significantly higher MAs levels in spleen and adrenal glands of male mice than in females in the 25-ppb iAs exposure group. There were no significant differences in tissue MAs levels from males and females in the 400-ppb iAs exposure group.

The relation between tAs and DMAs levels in tissues of Hs and WT mice were more complex than those found for the tAs and iAs or MAs levels. Statistically significant positive correlations between tAs and DMAs levels were found in tissues of Hs mice exposed to 25-ppb iAs and tissues of WT mice exposed to either 25- or 400-ppb (Fig. S3). Correlations was particularly strong in WT males and females after exposure to 400-ppb iAs. However, significant correlations between DMAs and tAs concentrations were not found for tissues of Hs mice exposed to 400-ppb. Analysis of tissue DMAs levels in Hs and WT mice identified both between- and within-genotype differences in levels of this iAs metabolite. For male mice exposed to 25-ppb iAs, DMAs concentrations were significantly higher in all tissues except kidney, liver, and adipose of WT males as compared to Hs males (Fig. 5). For the 400-ppb iAs exposure groups, DMAs levels were significantly higher in all tissues except adipose of WT males as compared to Hs males. In female mice exposed to 25-ppb iAs, DMAs levels were significantly higher only in muscle and heart of WT mice. For female mice, DMAs levels were significantly higher only in liver and adrenal glands of WT mice than in Hs mice from the 400-ppb exposure groups.

Within-genotype differences in tissue DMAs levels were found in both Hs and WT mice. In Hs mice in the 25-ppb iAs exposure group, DMAs levels in kidney and liver of males were significantly higher than in females. In contrast, DMAs levels in adrenal glands were significantly higher in females than in males. In WT mice in the 25-ppb iAs exposure group, DMAs levels in all tissues except muscle, adrenal glands, and adipose were significantly higher in males than in females. In Hs mice in the 400-ppb iAs exposure groups, adrenal DMAs levels were significantly higher in female than in males. In WT mice exposed to 400 ppb iAs, kidney DMAs levels were higher in males than in females. No other within-genotype differences in tissue DMAs levels were found in WT mice at this exposure.

### Proportions of arsenic species in tissues of WT and Hs mice

**Figure 6:**
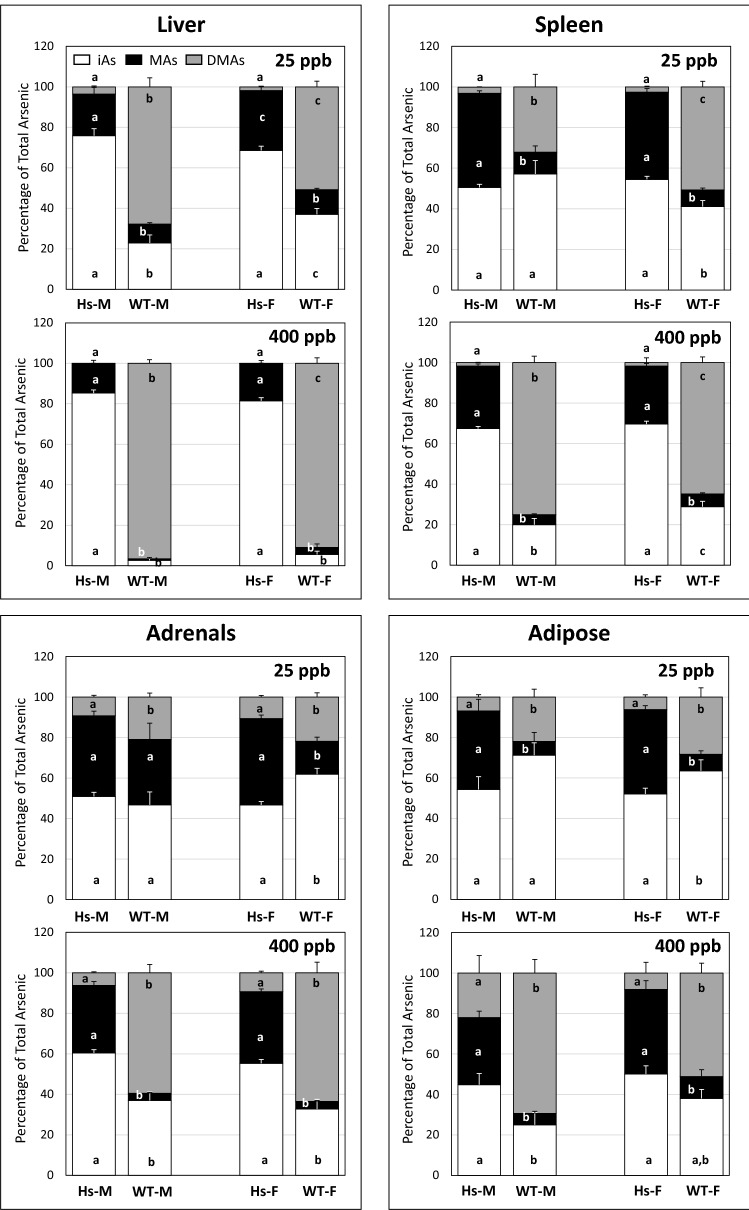
Percentages of total arsenic represented by inorganic arsenic (iAs), methylarsenic (MAs), and dimethylarsenic (DMAs) in liver, spleen, adrenals and adipose tissue of humanized (Hs) and wild-type (WT) male (M) and female (F) mice after 4-week exposure to 25-ppb or 400-ppb of inorganic arsenic in drinking water: The tissues of Hs mice with highest percentages of iAs and the corresponding tissues of WT mice are shown. Each bar and whisker represent mean and SEM value, respectively. For 25-ppb exposure, N = 6 for Hs males and WT males, and N = 8 for Hs females and WT females. For 400-ppb exposure, N = 7 and 8 for Hs males and WT males, respectively, and N = 10 and 11 for Hs females and WT females, respectively. For each arsenic species, values marked with different letters are significantly different (p < 0.05).

**Figure 7:**
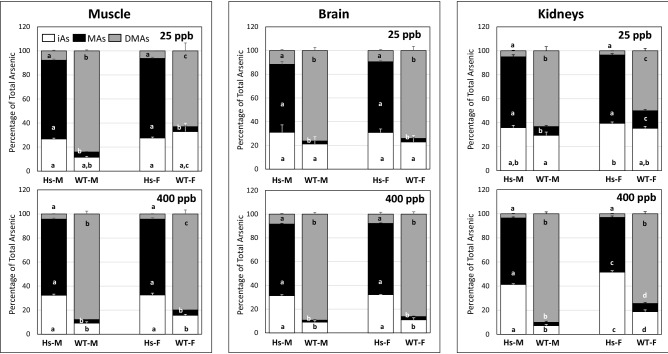
Percentages of total arsenic represented by inorganic arsenic (iAs), methylarsenic (MAs), and dimethylarsenic (DMAs) in muscle, brain and kidneys of humanized (Hs) and wild-type (WT) male (M) and female (F) mice after 4-week exposure to 25-ppb or 400-ppb of inorganic arsenic in drinking water: The tissues of Hs mice with highest percentages of MAs and the corresponding tissues of WT mice are shown. Each bar and whisker represent mean and SEM value, respectively. For 25-ppb exposure, N = 6 for Hs males and WT males, and N = 8 for Hs females and WT females. For 400-ppb exposure, N = 7 and 8 for Hs males and WT males, respectively, and N = 10 and 11 for Hs females and WT females, respectively. For each arsenic species, values marked with different letters are significantly different (p < 0.05).

**Figure 8:**
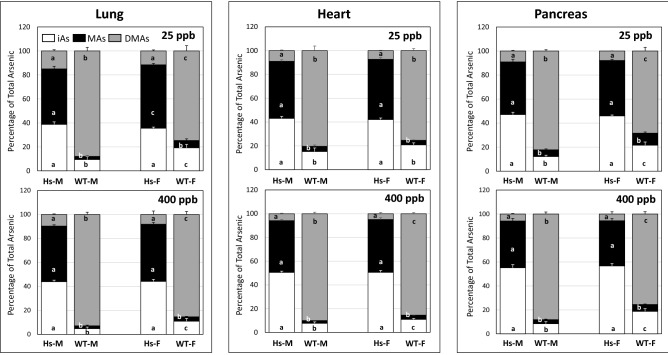
Percentages of total arsenic represented by inorganic arsenic (iAs), methylarsenic (MAs), and dimethylarsenic (DMAs) in lung, heart and pancreas of humanized (Hs) and wild-type (WT) male (M) and female (F) mice after 4-week exposure to 25-ppb or 400-ppb of inorganic arsenic in drinking water: The tissues of Hs mice with highest percentages of DMAs and the corresponding tissues of WT mice are shown. Each bar and whisker represent mean and SEM value, respectively. For 25-ppb exposure, N = 6 for Hs males and WT males, and N = 8 for Hs females and WT females. For 400-ppb exposure, N = 7 and 8 for Hs males and WT males, respectively, and N = 10 and 11 for Hs females and WT females, respectively. For each arsenic species, values marked with different letters are significantly different (p < 0.05).

Relative contributions of iAs and its methylated metabolites to tAs levels in tissues showed striking between-genotype variation (Figs. 6, 7, 8). In 25-ppb iAs exposure groups, % iAs was significantly higher in all tissues except kidney, spleen, adrenal, and brain in male Hs mice than in male WT mice. In female mice at this exposure level, %iAs was significantly higher in liver, spleen, lung, heart, and pancreas of Hs mice than in WT mice. In contrast, %iAs was significantly higher in adrenal glands and adipose from female WT mice than in female Hs mice. At the 400-ppb iAs exposure level, %iAs in all tissues of male and female Hs mice were significantly higher than in sex-matched WT mice. At both iAs exposure levels, tissue %MAs in male and female Hs mice were significantly higher than in sex-matched WT mice. In contrast, at both iAs exposure levels, tissue %DMAs in male and female WT mice were significantly higher than in sex-matched Hs mice.

### Concentrations of tAs in human tissues predicted by PBPK model

Concentrations of tAs in selected human tissues were estimated using a PBPK model^17^. This simulation assumed daily consumption of 2 L of water containing 25- or 400-ppb iAs for a period of 14 days. Table 1 shows predicted tAs concentrations in human tissues and measured tissue tAs levels in Hs and WT mice that consumed water containing the same iAs concentrations. At the 25-ppb exposure level, tAs levels predicted by the model were typically lower than tissue tAs concentrations in Hs mice and higher than tissue tAs concentration in WT mice. At the 400-ppb exposure level, predicted tissue tAs concentrations in humans were similar to those found in tissues of Hs mice. Notably, model-generated tissues tAs concentrations were typically closer to levels found in tissues of male Hs mice than tissue tAs levels for female Hs mice. Concentrations of tAs in tissues of WT mice exposed to 400-ppb iAs were about an order of magnitude lower than the corresponding model-generated values.

**Table 1:** Tissue total As concentrations (µg/kg) for humans and male and female Hs and WT mice exposed to 25- or 400-ppb (µg As/L) in drinking water for 14 days.

Exposure	Tissues	Humans^a^	Hs mice^b^		WT mice^b^	
			Males	Females	Males	Females
25 ppb	Kidneys	3.2	16.0 ± 2.1	15.3 ± 4.1	1.3 ± 0.6	0.9 ± 0.2
	Liver	7.2	12.6 ± 2.2	15.8 ± 4.8	0.7 ± 0.2	0.5 ± 0.1
	Muscle	2.1	3.7 ± 0.7	4.8 ± 1.5	0.8 ± 0.1	1.0 ± 0.4
	Lung	1.2	2.8 ± 0.5	3.5 ± 1.2	0.7 ± 0.2	0.6 ± 0.2
	Heart	2.1	2.6 ± 0.3	3.3 ± 0.8	0.6 ± 0.1	0.5 ± 0.1
	Brain	2.6	1.8 ± 0.3	2.2 ± 0.5	0.4 ± 0.1	0.3 ± 0.1
400 ppb	Kidneys	50.2	89.6 ± 29.1	141 ± 103.8	13.1 ± 7.2	7.5 ± 4.4
	Liver	118.5	91.6 ± 32.4	175.0 ± 92.6	5.2 ± 3.8	3.9 ± 3.9
	Muscle	33.1	31.5 ± 9.77	47.2 ± 20.1	7.6 ± 2.9	6.5 ± 4.4
	Lung	19.0	33.3 ± 13.3	72.3 ± 38.2	14.4 ± 8.0	12.4 ± 8.8
	Heart	33.1	27.4 ± 8.1	58.7 ± 33.8	6.7 ± 3.9	5.7 ± 3.5
	Brain	41.5	11.8 ± 3.8	23.0 ± 16.7	4.5 ± 2.0	3.5 ± 2.1

^a^Estimated steady state tAs levels calculated using the human PBPK model for iAs based on consumption of 2 L of iAs-contaminated drinking water per day.

^b^tAs levels in mouse tissues determined in present study (Mean ± SD, N = 6–11).

## Discussion

The role of arsenic methylation in the systemic distribution and clearance of arsenic has been demonstrated. Earlier work in mice showed that after exposure to iAs, MAs, or DMAs, these compounds and their metabolites were quickly cleared from tissues primarily by urinary excretion^26–29^. The linkage of arsenic methylation efficiency and the rate of clearance of arsenic has been demonstrated in studies in *As3mt* knockout mice in which loss of expression of *As3mt* markedly reduces capacity for methylation of iAs, leading to accumulation of iAs in tissues and a longer half time for whole body clearance of As^20,21^. Studies in human populations chronically exposed to iAs show that *AS3MT* polymorphisms that alter iAs methylation efficiency are associated with differences in susceptibility to a variety of diseases linked to iAs exposure^9,10,30–36^.

Because the toxic or carcinogenic actions of iAs have been linked to formation of its methylated metabolites, the significance of inter-species differences in iAs metabolism has been long recognized^7,37^. These differences between species in iAs metabolic efficiency are clearly exemplified by differences in the arsenic metabolic phenotype, the absolute and relative contributions of iAs, MAs, and DMAs to tAs excreted in urine. The most striking difference in arsenic metabolic phenotype between humans and other species is the much higher absolute and relative contribution of MAs to tAs in humans than in other species, including the laboratory mouse^38^. This interspecies difference in arsenic metabolic phenotype can reasonably attributed to underlying differences in the arsenic methylation genotype determined by kinetic factors (e.g., structural and functional differences in arsenic methyltransferases encoded in the genomes of different species) or to interspecies differences in the dynamic processes (e.g., protein binding or efflux pathways) that determine tissue concentrations of iAs and its methylated metabolites. The as yet unquantified effects of kinetic and dynamic differences in the fate of iAs in WT and Hs mice are reflected in differences in ratios of arsenical species in tissues and urine. Notably, these interspecies differences in arsenic metabolic efficiency in humans and other animal species may be a factor in mixed success of attempts to develop reproducible experimental models to study the toxic or carcinogenic effects of exposure to iAs and its methylated metabolites^7^.

Based on the putative relation between arsenic metabolic phenotype and arsenic methylation genotype described above, it was postulated that replacement of the *As3mt* gene of the laboratory mouse with human *AS3MT* gene would produce an organism in which the arsenic methylation phenotype resembled that found in humans. Thus, development of the Hs 129S6 mouse strain by syntenic replacement of the mouse *Borcs7/As3mt* locus with the human *BORCS7/AS3MT* locus permitted studies in the laboratory mouse of adverse effects of iAs exposure in context with a human-like iAs metabolism^18^. This work demonstrated clear differences in tissue and urinary levels of iAs and its methylated metabolites that were linked to differences in arsenic methyltransferase genotype. In addition, we found that expression of human *AS3MT* in livers of Hs mice was much lower than expression of *As3mt* in livers of WT mice. Because liver is thought to be a major site for the methylation of iAs^39,40^, differences in expression of the gene encoding the enzyme that catalyzes the methylation of iAs would be expected to change the pattern and extent of formation of MAs and DMAs. In contrast, *AS3MT* was highly expressed in adrenal glands of Hs mice, suggesting tissue-specific regulation of *AS3MT* expression in the presence of the human *BORCS7* gene. These differences in *AS3MT* and *As3mt* expression in Hs and WT mice are consistent with human data showing that sequence variation in the 10q24.32 region (which includes *AS3MT*) impacts expression of *AS3MT* in multiple tissues^41^.

The current research extends our earlier work with Hs mice to show the impact of expression of human *AS3MT* on levels of iAs and its methylated metabolites in tissues and urine after exposure to a high level of iAs in drinking water (400-ppb; Study 1) and a relatively low level of iAs in drinking water (25-ppb; Study 2). Use of the lower level for iAs exposure in the current research is germane to simulating exposures that occur in many populations worldwide that use iAs-contaminated water sources. Comparison of iAs metabolism at low and high exposure levels is particularly relevant, as it has been suggested that a high iAs exposure may result in saturation of the pathway for iAs methylation in humans, resulting in higher %iAs and %MAs, or lower %DMAs in urine^42–46^. Although this effect has not been observed in WT mice^14^, it was plausible to assume that saturation may occur in Hs mice, in which the low efficiency of iAs methylation resembles that in humans^18^. Indeed, we found that %iAs was significantly higher and %MAs was lower in urine of Hs mice exposed to 400-ppb iAs as compared to 25-ppb exposure. Although the differences in %DMAs were not statistically significant, the overall distribution of arsenic species in urine of Hs mice exposed to 400-ppb iAs is consistent with saturation of the pathway that converts iAs to DMAs. In contrast, in urine of WT mice %iAs was lower and %DMAs was higher after exposure to 400-ppb as compared to 25-ppb, suggesting possible stimulation of iAs methylation at the higher dose.

Our earlier report^18^ described concentrations and proportions of tAs and arsenic species in liver and kidneys of Hs and WT mice exposed to 400-ppb iAs in drinking water for 4 weeks (Study 1). Here, these data are compared with newly acquired data on distribution of arsenic species in urine and tissues of Hs and WT mice exposed to 25-ppb iAs (Study 2). Taken together, these data show that at either iAs exposure level, iAs methylation in Hs mice was much less efficient than in WT mice, leading to higher tAs concentrations and higher proportions of iAs and MAs in almost all tissues. Comparable tAs concentrations were found only in adrenal glands of WT and Hs males exposed to 25-ppb iAs. Notably, adrenal glands, lungs and kidney contained the highest tAs concentrations in WT mice exposed to either 25 or 400-ppb iAs. In contrast, in Hs mice, highest tAs levels were found in kidneys, liver and spleen.

Higher urinary %DMAs in women than in men has been reported, suggesting a sexual dimorphism in iAs methylation efficiency^42–49^. In the present study, we did not find significant sexual differences in tAs levels or in proportions of arsenic species in the urine of either Hs or WT mice at either iAs exposure level. However, the concentrations of tAs, iAs, and MAs in tissues of Hs females were higher than in Hs males; these differences were more common in the 400-ppb iAs treatment group. DMAs concentrations tended to be higher in tissues of Hs males. In WT mice at both iAs exposure levels, there were few between-sex differences in tissue tAs, iAs and MAs concentrations. Although DMAs concentrations were higher in tissues of WT males than in WT females, this occurred primarily at the 25-ppb iAs exposure level. These results suggest that Hs mice are an appropriate model for laboratory studies of the roles of sexual dimorphism in the metabolism and toxicity of iAs.

We previously applied the PBPK model to relate the concentrations of tAs found in liver and kidneys of Hs and WT mice exposed to 400-ppb iAs to tAs concentrations predicted for human tissues exposed to the same level of iAs in drinking water^18^. Here, we extended this comparison to other tissues supported by this model and included data from mice exposed to 25-ppb iAs. The comparison of the model-generated and experimental values suggests that, despite some differences, tAs concentrations in tissues of Hs mice matched better tAs concentrations predicted for human tissues, particularly for the 400-ppb exposure. The concentrations of tAs in tissues of WT mice exposed to 400-ppb were generally order of magnitude lower than those generated by the PBPK model and those found in tissues of Hs mice. Lung was an exception; tAs concentration in lung of WT mice matched better the predicted human concentrations than tAs in lungs of Hs mice. Model prediction of tissue tAs levels in mice exposed to a low level of iAs in drinking water (i.e., 25-ppb) may be confounded by the presence of iAs in diet. Even purified rodent diets, including AIN-93G rodent diet that was used in the present study, contains low ppb levels of iAs^49^. Thus, the aggregate of iAs from water and diet was likely underestimated in the modeling of tissue tAs levels. Similarly, in the absence of water supplies with elevated iAs levels, iAs in foods contribute significantly to aggregate iAs exposure in most human populations^2,50^ and ascertainment of aggregate intake of iAs from food and water may be difficult. For either species, uncertainty about the magnitude of iAs intake in low level exposure scenarios may affect the accuracy of model-generated tissues tAs.

## Methods

### Mice and treatments

The Hs 129S6 mice homozygous for the humanized *BORCS7/AS3MT* locus and co-isogenic WT 129S6 mice were used in both Study 1 and Study 2. Cohorts of mice used in these experiments were generated by intercross of Hs/WT heterozygotes. The WT and Hs littermates of the same sex were housed together after weaning under controlled conditions with 12-h light/dark cycle at 22 ± 1 °C and 50 ± 10% relative humidity.

#### Study 1

This study used a crossover design in which the same mice were used in two experiments. WT and Hs mice were initially used to examine metabolism and clearance of arsenic after a single dose of arsenite^18^. Here, a single oral dose of arsenite (20 µg As/kg of body weight) administered to 18–22 week old male and female WT and Hs mice was followed by urine and feces collection in metabolic cages for 72 h. Data on disposition of arsenic metabolites in urine and feces after the single dose of arsenite have been published^18^.

After completion of the single oral dose experiment, both Hs and WT mice were again caged together (mice of the same sex from one litter per cage) and fed AIN-93G purified rodent diet (Envigo Teklad, Madison, WI) for 5 weeks. During this time, all mice drank deionized water (DIW). This treatment allowed for clearance of arsenic from the body, as demonstrated by low levels of arsenic in urine (4 to 19 µg As/L) at week 5. Both WT and Hs mice (now 23 to 27 weeks old) were then exposed to iAs (sodium arsenite, 99% pure, Sigma-Aldrich, St. Louis, MO) in DIW (400 µg As/L) for 4 weeks. Spot urine samples (~ 50–100 µL) were collected weekly. To initiate urination, pressure was applied on mouse abdomen and drops of urine were collected by a pipette. Body weights were recorded before and after the exposure. After 4 weeks, all mice were euthanized by cervical dislocation without anesthesia and tissues were collected, including liver, kidneys, pancreas, spleen, heart, lung, adrenal glands, brain, visceral fat, and calf muscle.. All tissues were flash-frozen in dry ice and stored at − 80 °C.

#### Study 2

Five-week old male and female Hs and WT mice were fed a semi-purified AIN-93G (Dyets Inc., Bethlehem, PA) and drank deionized water (DIW) ad libitum for two weeks to minimize the body burden of arsenic accumulated from standard rodent diet which mice consumed in the mouse breeding colony. Notably, open-ingredient rodent diets often contain relatively high levels of arsenic which is present in a variety of inorganic and organic species^49^. As in Study 1, WT and Hs littermates of the same sex were housed together. All mice were then exposed to iAs (sodium arsenite) in DIW (25 µg As/L) for 4 weeks. After 4 weeks, spot urine samples were collected and all mice were sacrificed. Tissue samples were collected and stored as described for Study 1.

The methods were carried out in accordance with relevant guidelines and regulations. All procedures involving mice were approved by the University of North Carolina Institutional Animal Care and Use Committee. The study design and method description followed the ARRIVE guidelines, including use of WT mice as controls for the genetically modified Hs mice, use of WT and Hs mice from the same litter to minimize litter-to-litter variations, housing the WT and Hs in the same cage to minimize effects of coprophagy on studied outcomes, and using appropriate methods and to evaluate data generated by both studies. However, it should be noted that both WT and Hs mice exposed to 400-ppb iAs (Study 1) were older than the mice exposed to 25-ppb iAs (Study 2). In addition, mice in Study 1 received a single oral dose of iAs five weeks before sub-chronic exposure to 400-ppb in drinking water. Thus, age at exposure or prior exposure to iAs might affect the absolute and relative amounts of arsenic species in urine of mice exposed to 400 ppb.

### Analysis of arsenic species in urine and tissues

Speciation analysis of arsenic was carried out in spot urines and tissues collected at sacrifice. Tissues were homogenized in DIW using Wheaton Potter–Elvehjem style tissue grinders with PTFE pestle and Wheaton overhead stirrer apparatus (DWK Life Sciences, Milville, NJ). Ten percent tissues homogenates (w/v) and unfiltered urines were treated with 2% L-cysteine (Sigma-Aldrich) at room temperature for 1 h to reduce pentavalent arsenic species to their trivalent counterparts^51^. Most of the samples collected in Studies 1 and 2 were analyzed by hydride generation (HG)-cryotrapping (CT)-inductively coupled-mass spectrometry (ICP-MS), using Agilent 7500 or 7900 spectrometers (Santa Clara, CA) as the arsenic detector^52,53^. Spot urines, liver and kidneys collected in Study 1 were analyzed by HG-CT-atomic absorption spectrometry (AAS), using AAnalyst 800 spectrometer (Perkin-Elmer, Norwalk, CT). Both methods have been previously described^51–56^. This type of analysis determines concentrations of total iAs (iAs^III+V^), total MAs (MAs^III+V^) and total DMAs (DMAs^III+V^). Instrumental limits of detections (LODs) for iAs, MAs and DMAs analyzed by HG-CT-AAS are 14, 8 and 20 pg As, respectively^54^. LODs for HG-CT-ICP-MS analysis range from 0.27 to 1.7 pg As^52^. Total speciated arsenic (tAs) concentrations in urines and tissues were calculated as sum of iAs, MAs, and DMAs. Percentages of tAs represented by iAs (%iAs), MAs (%MAs), and DMAs (%DMAs) were calculated for urine and tissues to assess efficiency of iAs methylation.

### Statistical analysis

A non-parametric Kruskal–Wallis test with Dunn Multiple Comparisons was used to evaluate differences between genotypes and sexes in the concentrations of tAs in spot urine samples, which were not normally distributed. Differences in proportions of iAs, MAs and DMAs in urine and tissues of WT and Hs mice, which were found to be normally distributed, were evaluated using ANOVA and Student–Newman–Keuls Multiple Comparisons Test. Unpaired t-tests were used to compare tAs concentrations and concentrations of individual arsenic species in tissues of males and female mice of the same genotype, in WT and Hs mice of the same sex, and in mice of the same genotype and sex exposed to different levels of iAs (25- vs. 400-ppb). Linear regression and Spearman’s test were used to assess correlations between tAs concentration and concentrations of individual arsenic species in tissues. Differences and correlations characterized by p < 0.05 were considered statistically significant. InStat software package (GraphPad Software, San Diego, CA) was used for all statistical analyses.

### PBPK model

Concentrations of tAs in tissues of mice exposed 25 or 400 μg As/L were compared with tAs concentrations predicted for human tissues by a physiologically-based pharmacokinetic (PBPK) model^17^. This model consists of interconnected individual PBPK sub-models for iAs, MAs and DMAs. Each sub-model is constructed using flow limited compartments describing the mass balance of the chemicals in GI tract (lumen and tissue), lung, liver, kidney, muscle, skin, heart, and brain. Levels of tAs were obtained by adding the overall model-predicted concentrations of As, MAs, and DMAs in tissues assuming chronic exposure to 25 or 400 μg As/L and water consumption rate of 2 L/day for a period of 14 days.

## Supplementary Information

Supplementary Figures.

## Data Availability

Relevant data on tissue arsenic levels are provided in figures and the table in the manuscript and in the Supplementary Material.
