# Supplementary material for: Fate of arsenicals in mice carrying the human AS3MT gene exposed to environmentally relevant levels of arsenite in drinking water
Source: Sci Rep. 2023 Mar 4;13:3660. doi: 10.1038/s41598-023-30723-8 (PMC9985638; doi:10.1038/s41598-023-30723-8)
Supplement: Supplementary file 1 — Supplementary Figures. [file 41598_2023_30723_MOESM1_ESM.docx]

**SUPPLEMENTARY MATERIAL**

**Fate of arsenicals in mice carrying the human *AS3MT* gene exposed**

**to environmentally relevant levels of arsenite in drinking water**

Christelle Douillet^1^, Madison Miller^1^, Peter H. Cable^1^, Qing Shi^1^, Hisham El-Masri^2^, Tomáš Matoušek^3^, Beverly H. Koller^4^, David J. Thomas^5^, Miroslav Stýblo^1*^

^1^Department of Nutrition, University of North Carolina, Gillings School of Public Health, Chapel Hill, NC 27599, USA

^2^Chemical Characterization and Exposure Division, Center for Computational Toxicology & Exposure, Office of Research and Development, U.S. Environmental Protection Agency, Research Triangle Park, North Carolina 27709 USA

^3^Institute of Analytical Chemistry of the Czech Academy of Sciences, v. v. i., Veveří 97, 602 00 Brno, Czech Republic

^4^Department of Genetics, University of North Carolina, School of Medicine, Chapel Hill, NC 27599, USA

^5^Dinkey Creek Consulting, LLC, Chapel Hill, North Carolina 27517 USA

***Corresponding Author:**

Miroslav Stýblo, PhD

Department of Nutrition, Gillings School of Global Public Health

University of North Carolina at Chapel Hill

Chapel Hill, NC, 27599-7461, USA

Telephone: (919) 966-5721; Email: styblo@med.unc.edu

**Running Title:** Metabolism of inorganic As in mice expressing human *AS3MT*

**Disclaimer:** The Center for Computational Toxicology & Exposure, Office of Research and Development, U.S. Environmental Protection Agency has reviewed and approved this manuscript for publication. Approval does not signify that the contents reflect the views of the Agency, nor does mention of trade names or commercial products constitute endorsement or recommendation for use.

**Figure S1:** Correlation between average tAs and average iAs concentrations in liver, kidneys, pancreas, spleen, heart, lung, adrenal glands, brain, and visceral fat, calf muscle of male and female Hs and WT mice exposed to 25-ppb or 400-ppb iAs in drinking water. Linear regression lines, Spearman’s rank correlation coefficient (R_s_), and corresponding P values are shown.

**Figure S2:** Correlation between average tAs and average MAs concentrations in liver, kidneys, pancreas, spleen, heart, lung, adrenal glands, brain, and visceral fat, calf muscle of male and female Hs and WT mice exposed to 25-ppb or 400-ppb iAs in drinking water. Linear regression lines, Spearman’s rank correlation coefficient (R_s_), and corresponding P values are shown.

**Figure S3:** Correlation between average tAs and average DMAs concentrations in liver, kidneys, pancreas, spleen, heart, lung, adrenal glands, brain, and visceral fat, calf muscle of male and female Hs and WT mice exposed to 25-ppb or 400-ppb iAs in drinking water. Linear regression lines, Spearman’s rank correlation coefficient (R_s_), and corresponding P values are shown.
